# Description of the localized scleroderma subgroup of CARRAnet

**DOI:** 10.1186/1546-0096-10-S1-A71

**Published:** 2012-07-13

**Authors:** Eveline Wu, Egla C Rabinovich, Kathryn S Torok, Suzanne C Li, Robert C Fuhlbrigge, CARRAnet Investigators

**Affiliations:** 1 Brigham and Women's Hospital, Boston, MA, USA; 2 Duke University Medical Center, Durham, NC, USA; 3 Hackensack University Medical Center, Short Hills, NJ, USA; 4 University of Pittsburgh Medical Center, Pittsburgh, PA, USA

## Purpose

Localized scleroderma (LS) is a chronic inflammatory and fibrosing skin disease, more common in children. We present baseline data on the juvenile LS (jLS) cohort from the CARRAnet observational registry of pediatric rheumatologic disorders.

## Methods

We performed a cross-sectional baseline analysis of registry data for jLS.

## Results

Data were available on 44 children. 81.8% were female and 88.6% were Caucasian, of which 13.6% were Latino. Mean age at onset was 8.2 years (± 4.0), yet first evaluation by a pediatric rheumatologist was 9.9 years (± 4.2). Reported subtypes were: 34 linear scleroderma (LiScl: 25 trunk/limbs, 9 face/neck), 7 with circumscribed morphea (CM: 5 deep, 2 superficial), 6 with generalized morphea (GenM), 3 with eosinophilic fasciitis (EF), and 1 with pansclerotic morphea. There were 5 cases of mixed morphea (2 CM and LiScl, 1 with facial LiScl and GM, 2 with EF and linear lesions). Eight subjects had new lesions at time of enrollment. Features of active lesions included extension of existing lesions (13), warmth (13), erythematous/violacious color (13), and skin induration at lesion perimeter (10). Damage included subcutaneous atrophy (36), hyperpigmentation (35), dermal atrophy (31), hypopigmentation (19), hair loss (17), muscle atrophy (13), joint contracture (10), limb shortening (5), and hemifacial atrophy(1). Only three patients had extracutaneous manifestations, including two with arthritis. ANA positivity was found in 45% of tested patients, otherwise there were no consistent laboratory or imaging abnormalities. Table 1.

**Table 1:** 

	Antinuclear antibody	Elevated IgG	Eosinophilia	Abnormal aldolase	Abnormal creatine kinase	Abnormal CNS imaging	Abnormal GI study
Positive	14	6	5	3	1	1	3
Negative	17	20	30	16	24	10	3
Unknown	13	16	7	23	17	32	36
Total	44	42	42	42	42	43	42

Mean physician global assessment was 1.61 (range 0-8) and mean CHAQ score was 0.19 (0-1.13). On a visual analog scale (0-10), mean parent/subject score of overall well-being was 1.80 (± 1.66) and pain was 1.41 (±2.03). Health related quality of life was reported as excellent in 13, very good in 22, good in 7, and poor in 2 subjects. A worst ever and current ACR functional class > I was reported in 33% and 20.5%, respectively. Medications used are listed in table 2.

**Table 2:** 

	Oral methotrexate	Subcutaneous methotrexate	Mycophenolate mofetil	Intravenous corticosteroids	Longterm daily corticosteroids
Subjects (%)	21/42 (50%)	15/42 (36%)	15/42 (36%)	28-36 (78%)	17/35 (49%)
Current use	12	4	4	5	5
Past use	9	11	11	23	12

## Conclusion

jLS is reported more frequently in females and Caucasians in the CARRA Registry. LiScl is the most common lesion subtype, representing 77% of all patients. 45% of the jLS cohort is ANA positive. Subcutaneous and oral MTX, MMF, and pulse CS are the most common medications used for treatment. There is an almost 2 year delay in referral to pediatric rheumatology. There is significant morbidity associated with jLS with 30% reporting limitation in functional capacity.

## Disclosure

Eveline Wu: None; Egla C. Rabinovich: None; Kathryn S. Torok: None; Suzanne C. Li: None; Robert C. Fuhlbrigge: None; CARRAnet Investigators: None.

